# T Cell Recruitment in the Brain during Normal Aging

**DOI:** 10.3389/fncel.2012.00038

**Published:** 2012-09-19

**Authors:** Jickssa M. Gemechu, Marina Bentivoglio

**Affiliations:** ^1^Department of Neurological Sciences (DSNNMM), University of VeronaVerona, Italy

**Keywords:** lymphocytes, central nervous system, immunity, inflammation, blood-brain barrier, glia

## Abstract

Aging-related changes in the peripheral immune response are well documented, but less is known about changes of the immune response in the central nervous system. Reactivity of microglia, effectors of the brain innate immunity, is known to increase in the aged brain, but little attention has been hitherto devoted to T cell recruitment. Data in rodents point to a gradual enhancement of T cell homing to the brain in the steady state since the middle age. Experimental findings also point to enhanced transmigration of lymphocytes as part of an amplified response of the aging brain to acute exogenous inflammatory insults. Thus, available data support the capacity of the aged brain to mount a robust immune response, in contrast with peripheral immunity decline, and indicate that such central response involves recruitment of lymphocytes. These findings open many questions, including blood-brain barrier molecular regulation and infiltrated T cell subtypes during normal aging. The crosstalk between T cells, glia, and neurons also remains to be clarified in the aged brain parenchyma. This intercellular dialogue and related signaling could be relevant for both protection of the aged brain and its vulnerability to neurological disease.

## Introduction

The aging process starts, of course, at birth, but aging is in general referred to as the third part of the life of an individual, when age is advanced. All systems of the body are directly or indirectly affected by aging. Of special interest are different lines of evidence indicating that aging is associated with chronic, low grade inflammation, the so-called “inflammaging” (Franceschi et al., 2000), with a general increase in the production and release of proinflammatory mediators (see, for example, Krabbe et al., 2004). In the immune system, the state designated as immunosenescence affects the functions of innate and adaptive immunity (Desai et al., 2010). In the adaptive immune system, aging-associated remodeling of the T cell pool brings about a decrease of naïve T cells with increase of more differentiated memory T cells. This leads to multifaceted changes including a decline of the immune response (Desai et al., 2010; Shaw et al., 2010).

In the brain, which is an immune-specialized organ (Galea et al., 2007), aging-related variations of non-neuronal cells have been repeatedly reported. In particular, there is evidence of low grade microglial activation in the steady state (Conde and Streit, 2006; Lucin and Wyss-Coray, 2009). Microglia, the brain resident cells which belong to the myeloid lineage represent the innate immune response elements of the central nervous system (CNS). Microglia are efficient sensors of changes in the CNS microenvironment, and their neuroprotective role has been hypothesized to be impaired during aging (Streit and Xue, 2012).

Much less information is instead available on the migration of circulating T cells into the aging CNS. Interest in lymphocyte extravasation into the CNS parenchyma has been traditionally linked to studies on multiple sclerosis and its animal model, experimental autoimmune encephalomyelitis, in which the entry of T cells, and in particular CD4+ T helper (Th) cells, into the CNS plays a key pathogenetic role (Engelhardt and Sorokin, 2009). CD4+ Th1 cells produce the proinflammatory cytokines interleukin (IL)-2, interferon (IFN)-γ, and tumor necrosis factor (TNF)-β, promoting inflammation (Aloisi et al., 2000). Multiple sclerosis typically affects young adults (see, for example, Koutsouraki et al., 2010), so that T cell recruitment to the brain during aging has not received much attention. In recent years, however, interest on lymphocytes in the aged CNS has been stimulated by reports of the occurrence of these cells, though in small numbers, in the brain affected by neurodegenerative diseases, including Parkinson’s disease and Alzheimer’s disease (AD) (Lucin and Wyss-Coray, 2009; Rezai-Zadeh et al., 2009).

Knowledge of brain changes during normal aging is a prerequisite to an understanding of alterations during disease. Neurological diseases for which aging represents a risk factor, and AD in particular, are not “inevitable consequences” (Nelson et al., 2011) of aging *per se*. However, the fact that susceptibility to specific pathways leading to these diseases increases at an advanced age implicates changes in the brain which remain to be clarified.

In this context, the present brief overview is focused on T cells transmigration to the aging brain under normal conditions and in response to acute inflammatory challenges.

## T Cells in the Healthy Aged Brain

### The aging blood-brain barrier: An experienced sentinel or a leaky door?

**Figure 1:**
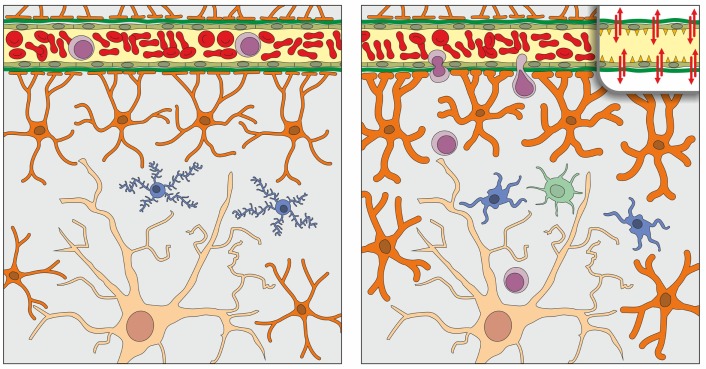
**Schematic representation of the normal organization of the parenchyma and blood-brain barrier (BBB) of the young adult (left) and aged (right) brain, as derived from studies that are here reviewed**. In the brain parenchyma, a single neuron (light brown), astrocytes (orange), and microglia (blue) are represented; oligodendrocytes and perivascular macrophages are not shown. Astrocytic endfeet contribute to the formation of the BBB, together with tight junctions of endothelial cells of cerebral microvessels and their respective basement membranes (represented for simplicity as a single green band). In the aged brain compared to the young brain, astrocytes (depicted as hypertrophic) and microglia (hypertrophic with stout, less ramified processes) show a low grade activation in the steady state; in addition, as shown in the inset (upper right) tight-junctional assemblies between endothelial cells may be altered. At variance with the young brain in which T cell (purple) entry is restricted by the BBB, T cells can infiltrate the aged brain parenchyma in low numbers. Access to dendritic cells (one dendritic cell is shown in light green) may also be facilitated in the aged brain. Adapted and modified from Bentivoglio et al. (2011).

The blood-brain barrier (BBB) is a specialized system of the endothelial cells of cerebral microvessels which express unique intercellular tight junctions and are associated with a basement membrane (Figure 1). The BBB also includes pericytes, perivascular macrophages, ensheathing astrocytic endfeet, and associated parenchymal basement membrane. This barrier shields the brain from the blood milieu, and acts as very selective filter for molecules and cells (Bechmann et al., 2007; Engelhardt and Sorokin, 2009). The BBB thus plays an important role in the maintenance of the brain microenvironment, and represents a key component of the so-called neurovascular unit (Hawkins and Davis, 2005).

Structural and functional alterations of the BBB have been reported in the normal aging brain, including alterations of carrier-mediated transport mechanisms (Shan and Mooradian, 1997). The findings on aging-related variations of BBB permeability are, however, not unequivocal. Based on the cerebrospinal fluid/serum ratio of albumin, it has been reported that BBB permeability is increased in normal aging (reviewed in Popescu et al., 2009), and such alterations have been involved in the development of cerebral microvascular disease in humans (Farrall and Wardlaw, 2007). Recent findings in the aged rodent brain also support an aging-dependent increase of BBB permeability, which may show regional differences (Bake et al., 2009; Blau et al., 2012; Lee et al., 2012). However, other studies in humans have suggested that BBB alterations are very subtle throughout adult life, or could even be related to subclinical disease (see, for example, Blennow et al., 1991; Garton et al., 1991; Skoog et al., 1998) and thus spare the non-diseased brain.

The full spectrum of aging-related changes in the molecular composition of tight junctions in the aged CNS endothelia still has to be unraveled. Cerebral occludin content was found to be significantly reduced in 24-month-old rats, but not in mice of the same age, whereas zonula occludens-1 protein did not show significant age-related differences in 24-month-old rats and mice (Mooradian et al., 2003; Lee et al., 2012). In isolated brain microvessels, claudin-5 protein distribution in tight junction assemblies was found to be disrupted in reproductive senescent female rats and post-menopausal women at variance with age-matched male samples, indicating that claudin-5 regulation could be sensitive to persistent changes of ovarian hormone cyclicity (Bake et al., 2009). Claudin-5 transcript levels have been reported to be reduced in the aged mouse brain following injury caused by cortical contusion (Lee et al., 2012). Specific tight-junctional proteins may, therefore, show aging-related and hormone-related changes, but such variations do not seem to involve the entire repertoire of these proteins. Matrix metalloproteinases (MMPs), secreted by activated T cells and macrophages, can degrade extracellular matrix components serving as effectors of inflammatory responses including cell migration and tissue remodeling (Larochelle et al., 2011). Increased baseline MMP activity was found in the aged mouse brain, but only MMP-9 showed increased activity following cortical contusion (Lee et al., 2012).

Further knowledge on physiological changes of the BBB over lifetime is needed, but the available data indicate that the BBB function could be largely preserved during aging, though with alterations which may especially impair the brain response to different kinds of insult.

### T cells in the aging brain

The BBB restricts access of leukocytes into the CNS; more in general, the entry of monocytes, B cells, and T cells into the brain parenchyma is very tightly regulated (Bechmann et al., 2007). Immune cell migration across the BBB appears to be mainly controlled by the expression of adhesion molecules, chemokines and their receptors. Under normal conditions the entry of antigen-activated lymphocytes is regulated especially by the restricted expression of cell adhesion molecules in CNS endothelia (Greenwood et al., 2011), which is a main feature of the immune specialization of the CNS (Bechmann et al., 2007). In particular, intercellular adhesion molecule (ICAM)-1 plays a key role in the recruitment of T cells in the CNS (Greenwood et al., 2011).

The barrier represented by endothelial tight junctions is especially tight in cerebral microvessels. Leukocyte transmigration, which is a multistep process, occurs mainly at postcapillary venules and is likely to involve both a paracellular route and transcellular diapedesis (Bechmann et al., 2007; Greenwood et al., 2011). T cell entry can in turn activate endothelial cells through the release of leukocyte-derived cytokines, which can induce or upregulate the expression of cell adhesion molecules (Greenwood et al., 2011). Release of proinflammatory cytokines such as IL-1β and TNF-α by activated glia in the brain, or their presence in the blood, also leads to an upregulation of the expression of cell adhesion molecules on microvascular endothelial cells (Weng, 2006). These mechanisms amplify the inflammatory cascade, and could be enhanced in the aging brain due to its proinflammatory phenotype. Passage of T cells into the brain is also facilitated by chemokines secreted by reactive astrocytes, including monocyte chemotactic protein (MCP)-1 and macrophage inflammatory protein-1 (MIP)-1α (Aloisi et al., 2000). It is of interest to note that the expression of these chemokines increases in the brain during aging (Kumagai et al., 2007; Blau et al., 2012).

In young adulthood, given the restricted access of immune cells to the CNS, T cells are rare in the brain parenchyma and are found in the meninges and choroid plexus (Hickey, 2001). Experimental data indicate, however, that lymphocyte extravasation into the brain parenchyma can moderately increase with advancing age.

T cell occurrence in the brain has been examined in mice of different age groups (3–4, 6, 12, and 24–30 months of age) by immunophenotyping with the pan-T cell marker CD3 (Stichel and Luebbert, 2007). In this study, T cells have been identified in the brain parenchyma of 12-month-old and older mice, with an aging-related increase in number and accumulation in the white and gray matter. Concomitant features of activation of astrocytes and microglia with marked increase of IL-1β and TNF-α immunoreactivities were also noted (Stichel and Luebbert, 2007). Dendritic cells, immunostained by the myeloid dendritic marker CD11c, also showed an aging-dependent accumulation in the brain (Stichel and Luebbert, 2007). Dendritic cells, the professional antigen-presenting cells which initiate the adaptive immune response providing a bridge with adaptive immunity, can be detected in the meninges and choroid plexus under normal conditions and are rarely found in the healthy brain parenchyma of young subjects (Bechmann et al., 2007; Zozulya et al., 2010).

T cell trafficking has also been investigated in the retina, the outpost of the brain sealed by the blood-retinal barrier, comparing adult (3- to 6-month-old) and aged (24- to 30-month-old) rats (Chan-Ling et al., 2007). Cells immunopositive for the T cell receptor have been identified in the superficial plexus of the retina of the aged rats, together with other inflammatory features, such as major histocompatibility antigen class II immunopositivity, which was not observed in the retina of the young rats (Chan-Ling et al., 2007).

To the best of our knowledge, no systematic analysis of T cell infiltration in the non-diseased human brain at different ages has been pursued. However, in the postmortem study of the hippocampus of three human cases without neurological disease (69, 74, 77 years of age, respectively), used as controls of AD brains, T cells have been identified in variable numbers, though in lower number than in the hippocampus of severe AD cases (Togo et al., 2002).

## Inflammation-Induced T Cell Recruitment to the Aging Brain

Experimental data indicate that responses to inflammatory stimuli in the brain are not only preserved but also include a gradual amplification with advancing age.

In particular, investigations in our laboratory on the response to the intracerebroventricular (icv) administration of the proinflammatory cytokines IFN-γ and TNF-α, separately or in combination, have determined that the acute response of astrocytes and microglia to cytokine exposure is markedly enhanced in mice of 18–21 months of age with respect to young mice, without the occurrence of neurodegenerative events (Deng et al., 2006). Interestingly, a study of apoptosis-regulatory proteins in the brain of aged mice after central cytokine exposure has pointed out a protective response with enhancement of anti-apoptotic protein upregulation compared to young mice (Xu et al., 2007).

An amplified central innate immune response to inflammatory challenges, with enhanced proinflammatory profile has also been reported in other studies on aged rodents (for review, see Wynne et al., 2009; Bertini et al., 2010). For example, icv administration of the endotoxin lipopolysaccharide has been reported to enhance the expression of transcripts of proinflammatory cytokines and sickness behavior in 22- to 24-month-old mice (Huang et al., 2008). T cell entry was not, however, examined in these studies. Enhanced inflammation seems to exert a detrimental effect on cognitive abilities in aged rodents (reviewed by Lynch, 2010).

The expression of transcripts involved in T cell homing and in cytokine signaling was examined in our laboratory in the septum and hippocampus of aged (20- to 24-month-old) *versus* young (2- to 3-month-old) mice after icv injection of IFN-γ or TNF-α (Xu et al., 2010). In addition, the expression of ICAM-1 and the magnitude of T cell infiltration was examined in several brain regions of mice of three age groups (3–4, 8–9, 15–16 months of age) at 48 h after icv injection of TNF-α (Xu et al., 2010). The main findings pointed out that the expression of transcripts encoding suppressor of cytokine signaling (SOCS) molecules 1 and 3, ICAM-1, MMP12, and the chemokine CXCL9 was preserved in the aged brain after cytokine exposure. ICAM-1 immunoreactivity and protein content showed instead a progressive age-related upregulation in response to TNF-α, which was evident not only in microglia, but also in astrocytic endfeet, i.e., the astrocytic compartment specifically involved in barrier function. Interestingly, a gradual age-related increase of the number of T cells in the brain parenchyma and choroid plexus was found after central cytokine exposure (Xu et al., 2010). At an earlier time point (6 h) after injections of IL-1β or TNF-α into the striatum of 18-month-old rats, increased accumulation of brain neutrophils and macrophages was found, but no age-related difference was reported in the number of cytokine-dependent T cells (Campbell et al., 2007), suggesting that longer time intervals are needed for the amplification of this event.

In these paradigms, T cells have been observed sparsely scattered in the brain parenchyma besides a perivascular distribution, thus indicating a migratory capacity.

It is worth mentioning here that leukocyte infiltration in the brain occurs after central (icv or intracerebral) but not after peripheral (such as intravenous or intraperitoneal) administration of inflammatory mediators (Proescholdt et al., 2002), although both central and peripheral routes of administration increase proinflammatory molecules in the brain. This indicates that the abluminal side of cerebral microvessels should be activated for T cell entry.

As a further note on injury-related responses of T cells during aging, it is of interest that in the model of axonal injury represented by transection of the facial nerve, in which T cells infiltrate the axotomized facial motor nucleus, such response was found to be amplified in 12-month-old mice with respect to 3-month-old ones (Dauer et al., 2011).

## T Cell Crosstalk with Resident Cells of the Aged Brain

Several findings indicate that T cells need to be activated to cross the BBB (Aloisi et al., 2000). Imaging data with two-photon microscopy have confirmed that naïve T cells are unable to migrate within the healthy, non-inflamed brain parenchyma (Herz et al., 2011). The low grade inflammatory status of the aged brain parenchyma could favor their recruitment, but the critical mediators of this phenomenon, and the T cell subtypes entering an aged brain remain to be established. The phenotyping of T cell populations after acute inflammatory challenge has indicated that both CD4+ and CD8+ T cells can infiltrate the aged mouse brain (Xu et al., 2010). This, however, is not sufficient to determine the full extent of lymphocyte subsets (including Th1 and Th2, Th17, T regulatory cells), which can infiltrate the aged brain parenchyma in the steady state and in inflammatory conditions.

The dialogue of T cells with glia and neurons in the aged brain also requires to be clarified. The functional outcome of T cell homing toward neuroprotective or detrimental effects depends from an array of interactions of T cells with glial cells and neurons. Interaction between T cells and microglia can exert an impact on the activation state of either cell type (Aloisi et al., 2000; Lynch, 2010). CNS-infiltrating Th1 cells stimulate microglia, which can in turn regulate the balance between Th1 and Th2 responses; Th2 cells, which produce IL-4, IL-10, and IL-13, may inhibit Th1-induced inflammation, limiting its detrimental effects (Aloisi et al., 2000). This network of interactions, which also involves astrocytes, can be very critical in the aged brain in view of the steady state activation of glial cells.

A wealth of data point to a neuroprotective effect of T lymphocyte infiltration in the brain (Schwartz and Shechter, 2010), probably as a result of T cell-produced neurotrophins, but activated T cells may also inhibit or promote neuroprotective glial responses (Carson et al., 2006). It is noteworthy, in this respect, that microglia – T cell interactions can skew a detrimental or protective response, and that circulating leukocytes have been related to brain plasticity and cognitive abilities (Schwartz and Shechter, 2010). The beneficial and deleterious effects of peripheral immune cell trafficking into the brain can also depend on disease state, and this could be especially relevant in neurodegenerative diseases (Rezai-Zadeh et al., 2009).

## Conclusion and Perspectives

Taken together the findings that have been here reviewed indicate that the inflammatory phenotype of the aged brain parenchyma includes a moderate number of infiltrated lymphocytes in the steady state (Figure 1). In addition, T cells are players in the gradual amplification of the brain immune response with advancing age. This could be due to an increase of mediators responsible for T cell entry. However, as highlighted above, many questions remain open. Among these, the immunological function of T cells which infiltrate the aged brain and their state of activation, as well as the responsible molecular changes in the BBB require clarification. The effect of leukocytes on the proinflammatory milieu and guidance of T cell migratory behavior also remain to be investigated in the aged brain.

Enhanced T cell homing could expose the aged brain to detrimental effects of bystander inflammation, but could also represent an adaptation/compensation to the aging-related reduced T cell function. Furthermore, infiltrated T cells could meet the demand of an increased immune surveillance to protect aged neurons “primed” by lifelong attacks to their precious and unique function.

## Conflict of Interest Statement

The authors declare that the research was conducted in the absence of any commercial or financial relationships that could be construed as a potential conflict of interest.
